# Fengycin Produced by *Bacillus amyloliquefaciens* FZB42 Inhibits *Fusarium graminearum* Growth and Mycotoxins Biosynthesis

**DOI:** 10.3390/toxins11050295

**Published:** 2019-05-24

**Authors:** Alvina Hanif, Feng Zhang, Pingping Li, Chuchu Li, Yujiao Xu, Muhammad Zubair, Mengxuan Zhang, Dandan Jia, Xiaozhen Zhao, Jingang Liang, Taha Majid, Jingyuau Yan, Ayaz Farzand, Huijun Wu, Qin Gu, Xuewen Gao

**Affiliations:** 1 Department of Plant Pathology, College of Plant Protection, Nanjing Agricultural University, Key Laboratory of Integrated Management of Crop Diseases and Pests, Ministry of Education, Nanjing 210095, China; rao.alvina@yahoo.com (A.H.); 2017202058@njau.edu.cn (F.Z.); 2017102044@njau.edu.cn (P.L.); 2018102014@njau.edu.cn (C.L.); 2018102015@njau.edu.cn (Y.X.); Zubair_biotech@yahoo.com (M.Z.); 12115229@njau.edu.cn (M.Z.); 2017802180@njau.edu.cn (D.J.); 2016202006@njau.edu.cn (X.Z.); tahamajid1705@yahoo.com (T.M.); 12115231@njau.edu.cn (J.Y.); ayaz.farzand@uaf.edu.pk (A.F.); hjwu@njau.edu.cn (H.W.); 2 Development Center of Science and Technology, Ministry of Agriculture and Rural Affairs, Beijing 100176, China; liangjingang@agri.gov.cn

**Keywords:** fungal-bacterial interactions, *Bacillus amyloliquefaciens*, *Fusarium graminearum*, Fengycin, mycotoxins

## Abstract

*Fusarium graminearum* is a notorious pathogen that causes Fusarium head blight (FHB) in cereal crops. It produces secondary metabolites, such as deoxynivalenol, diminishing grain quality and leading to lesser crop yield. Many strategies have been developed to combat this pathogenic fungus; however, considering the lack of resistant cultivars and likelihood of environmental hazards upon using chemical pesticides, efforts have shifted toward the biocontrol of plant diseases, which is a sustainable and eco-friendly approach. Fengycin, derived from *Bacillus amyloliquefaciens* FZB42, was purified from the crude extract by HPLC and further analyzed by MALDI-TOF-MS. Its application resulted in structural deformations in fungal hyphae, as observed via scanning electron microscopy. In planta experiment revealed the ability of fengycin to suppress *F. graminearum* growth and highlighted its capacity to combat disease incidence. Fengycin significantly suppressed *F. graminearum*, and also reduced the deoxynivalenol (DON), 3-acetyldeoxynivalenol (3-ADON), 15-acetyldeoxynivalenol (15-ADON), and zearalenone (ZEN) production in infected grains. To conclude, we report that fengycin produced by *B. amyloliquefaciens* FZB42 has potential as a biocontrol agent against *F. graminearum* and can also inhibit the mycotoxins produced by this fungus.

## 1. Introduction

China is the leading producer of wheat worldwide, and *Fusarium graminearum* is a major causal agent of Fusarium head blight (FHB) epidemics in the country, affecting various cereal crops, either in the field or upon their storage in humid conditions [1,2]. Infections in the field can occur at any stage, from anthesis to kernel development, and this plant pathogenic fungus mainly infects florets. Under favorable environmental conditions, an infection can be established within 3 to 4 days. *F. graminearum* produces numerous potentially important mycotoxins. Deoxynivalenol (DON) is the most abundant form of the trichothecenes found in grain and is a sesquiterpenoid. Acetylated derivatives of DON, less toxic than DON, are also found in grains 15-ADON. While trichothecenes are known to be produced during the early stages of the infection process in host plants, the most common non-steroidal estrogenic mycotoxins and Zearalenol (ZEN) is produced at the end of the infection process [3]. Fungus invades and colonizes grains and produces deoxynivalenol [4], which is the most common mycotoxin. Infected seeds show reduced germination and produce weaker seedlings. DON is the final product of the trichothecene biosynthetic pathway. It causes several biological disturbances and acts as an inhibitor during protein synthesis [5]; moreover, it is highly toxic, and thus unfit for the consumption of humans or animals [1,6]. FHB management remains challenging. There are still very few varieties of wheat that are highly resistant to *F. graminearum*. Synthetic chemicals are effective for controlling FHB in wheat; however, they are inevitably associated with environmental pollution and resistance development in *F. graminearum* [7]. Therefore, to control FHB in wheat, it is of high urgency to explore alternative management strategies that are not only reliable but also less toxic to the environment.

To date, biocontrol agents have attracted huge scientific attention as they are environmentally friendly [8]. Plant growth-promoting rhizobacteria (PGPR) are evidently promising for suppressing various fungal diseases and stimulating plant growth. Many PGPR strains have been successfully formulated as biopesticides to control plant diseases [9]. *Bacillus* spp. are the most promising antagonistic PGPR. *Bacillus amyloliquefaciens* FZB42 (now called *B. amyloliquefaciens* subsp. *plantarum* FZB42) is a Gram-positive strain and well known for its antagonistic activity, extensive rhizosphere colonization, and plant growth stimulation [10,11]. This strain reportedly produces secondary metabolites that suppress soil-borne plant pathogens; genome analysis of FZB42 revealed 10 gene clusters, covering nearly 10% of the whole genome, and these are responsible for producing secondary metabolites that display antimicrobial and nematocidal activities. These secondary metabolites include three lipopeptides (surfactin, bacillomycin D, and fengycin), three polyketides (macrolactin, bacillaene, and difficidin) [3,4], one siderophore (bacillibactin), one antibacterial dipeptide (bacilysin), and two ribosomally produced and post-translationally modified peptides plantazolicin and amylocyclicin. FZB42 can also synthesize plant hormones, such as indole-3-acetic acid, and produce volatile compounds, such as 2,3-butanediol, to promote plant growth. All these metabolites contribute to the biocontrol properties of FZB42. Moreover, this strain displays strong antagonistic activity against fungi, such as *Rhizoctonia solani*, *Botrytis cinereal* [6], *F. oxysporum* [7], and against bacteria, such as *Erwinia amylovora* [8] and *Xanthomonas oryzae* [12]. A recent study demonstrated that bacillomycin D is involved in antagonistic interactions with *F. graminearum*, provoking physiological and metabolic changes during the antagonism [13]. The living spores of FZB42 have also been used to develop commercial products, such as RhizoVital^®^. Accordingly, FZB42 seems to be a good candidate for use as a biocontrol agent against plant pathogens in agricultural production systems.

Here we report that fengycin produced by *B. amyloliquefaciens* FZB42 significantly inhibits the growth of *F. graminearum* and the biosynthesis of the mycotoxins, including deoxynivalenol (DON), 3-acetyldeoxynivalenol (3-ADON), 15-acetyldeoxynivalenol (15-ADON), and zearalenone (ZEN).

## 2. Results

### 2.1. Fengycin Produced by B. amyloliquefaciens FZB42 mutant AK1S Displayed Antagonistic Activity Against F. graminearum

**Figure 1:**
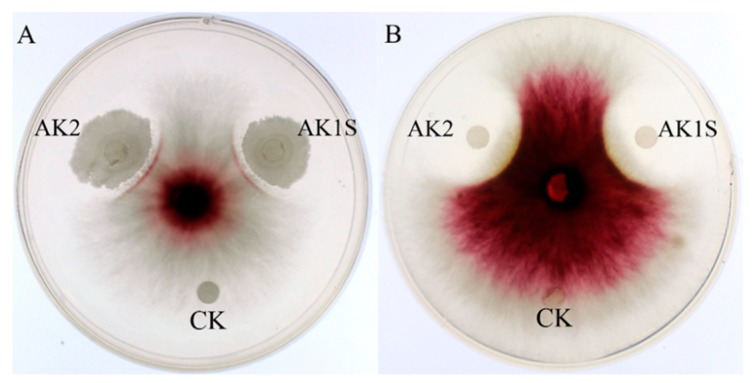
Antagonistic activities of AK2 and AKIS against *F. graminearum* PH-1 (**A**) and of their secondary metabolite extract (**B**). CK, control (LB medium or methanol).

It has already been reported that both *B. amyloliquefaciens* FZB42 and its crude extract of secondary metabolites could suppress *F. graminearum* growth. The mutant AK2 and AK1S cultures and their crude extracts showed inhibition activity against *F. graminearum* growth, as indicated by clear zones in the inoculated bacteria and extracts (Figure 1).

**Figure 2:**
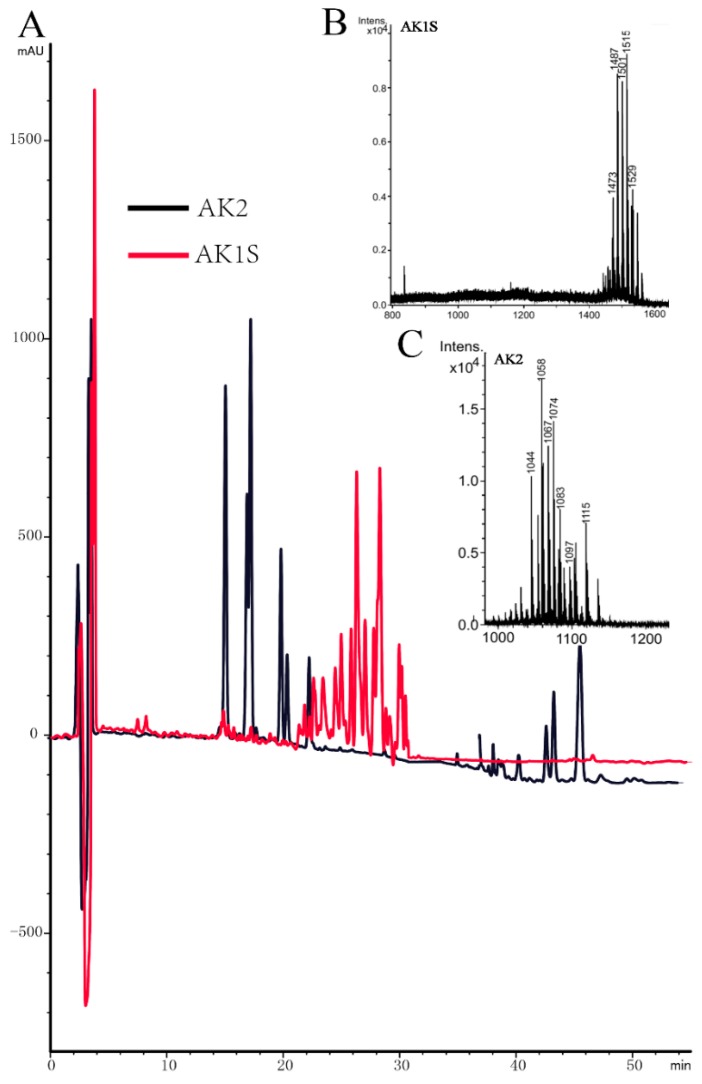
Analysis of lipopeptides produced by AK2 and AKIS using HPLC (**A**) and MALDI-TOF-MS (**B**,**C**).

AK2 could produce bacillomycin D and surfactin, but not fengycin; HPLC results indicated that AK2 showed typical peaks for bacillomycin D (from 16 to 20 min) and surfactin (from 40 to 48 min) (Figure 2). MALDI-TOF-MS analysis also confirmed that AK2 could only produce bacillomycin D and surfactin. There were peaks (M + H)^+^ for molecular ion peaks (M + Na)^+^ for C_14_–C_15_ surfactin at *m*/*z* 1044 and 1058, and ion peaks (M + K)^+^ for C_15_ surfactin at *m*/*z* 1074 (Figure 2). Furthermore, there were molecular ion peaks (M + Na)^+^ for C_15_ bacillomycin D at *m*/*z* 1067, and ion peaks (M + K)^+^ for C_15_–C_16_ bacillomycin D at *m*/*z* 1083 and 1097 (Figure 2). 

Among these molecules, all containing the same ion, there was a 14-Da difference in molecular weight, suggesting the presence of varying lengths of fatty acid chains within bacillomycin D (CH2 = 14 Da). AK1S, containing double mutation, could produce fengycin, but not bacillomycin D or surfactin, as confirmed via HPLC analysis, which showed peaks only for fengycin (from 24 to 30 min). MALDI-TOF-MS confirmed this result. There were molecular ion peaks (M+K)^+^ for Ala-6-C_14_–C_18_ fengycin at *m*/*z* 1473, 1487, 1501, 1515, and 1529 (Figure 2). Our results indicated that both fengycin and bacillomycin D act as fungicidal factors and cause in vitro suppression of *F. graminearum* growth. This result coincides with the results of our previous study [13].

### 2.2. Ultrastructural Changes Caused by Fengycin in F. graminearum Hyphae

**Figure 3:**
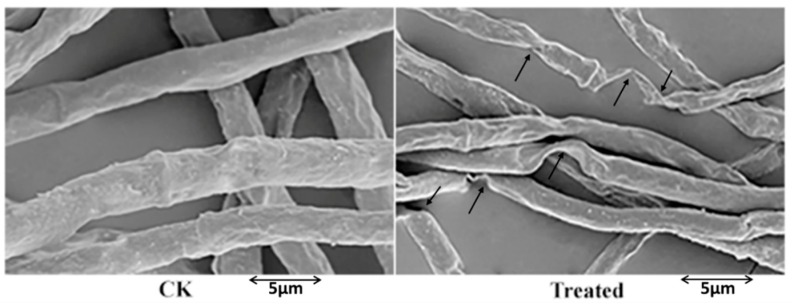
Effect of fengycin (90 µg/mL) on *F. graminearum* hyphae. *F. graminearum* hyphae were treated with pure fengycin, and electron micrographs were obtained. The hyphal morphology of *F. graminearum* was altered by fengycin; in comparison with the control, several deformed hyphal structures were observed in the fengycin-treated sample. Arrowheads indicate abnormal morphology of PH-1 hyphae. Bar: 5 µm.

To elucidate the mechanism by which fengycin affects *F. graminearum* hyphal growth, we observed the morphological variations in fungal mycelia using scanning electron microscopy [14]. The micrographs showed that fengycin triggered a range of abnormalities in *F. graminearum* hyphae; fengycin-treated hyphae showed considerable deformation—they were thin and twisted, and some parts along the hyphal walls were ruptured (Figure 3). On the other hand, the micrographs of the untreated control demonstrated healthy, dense, and cylindrical hyphae.

### 2.3. Fengycin Reduced F. graminearum Pathogenicity and Mycotoxins Biosynthesis

**Figure 4:**
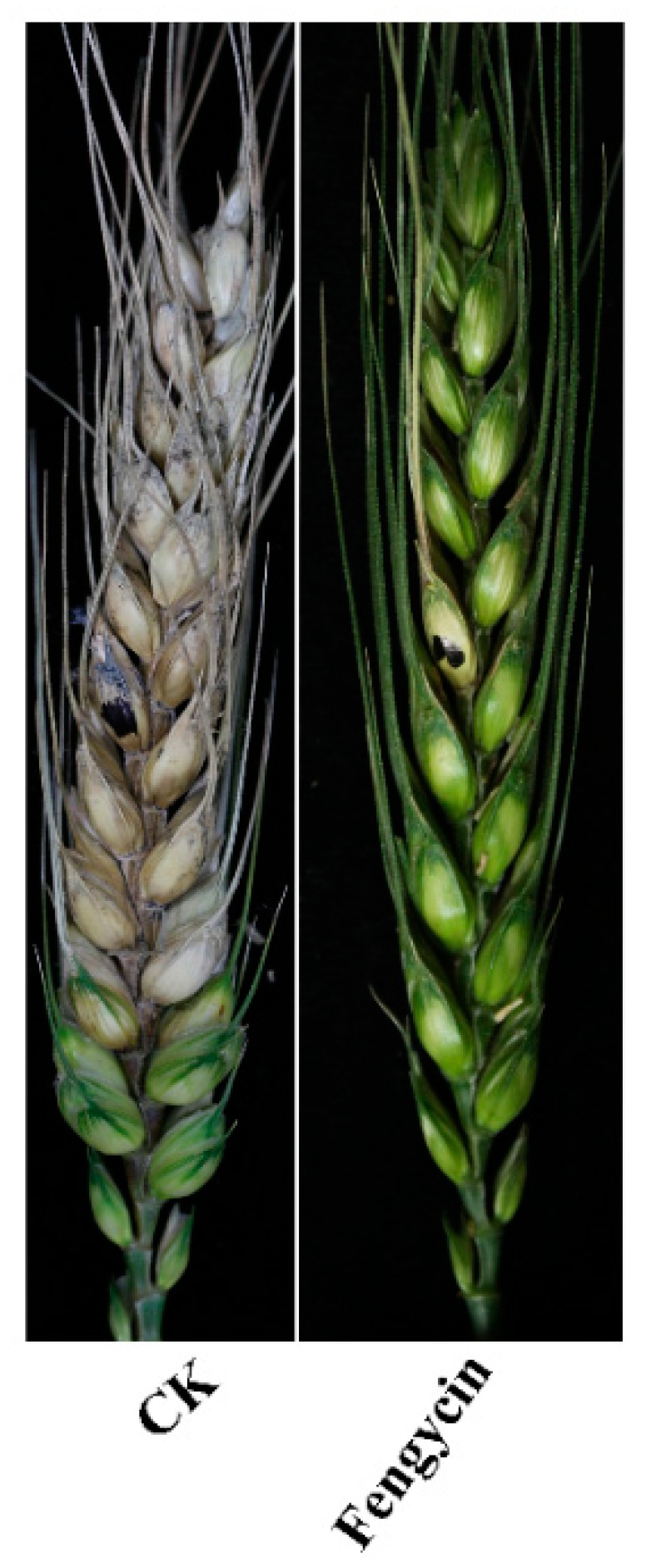
Effects of fengycin on pathogenicity of *F. graminearum*. Wheat heads were drop inoculated with conidial suspensions of *F. graminearum* PH-1 and then were treated with 90 µg/mL fengycin. Conidial suspension with 6.67% (*v*/*v*) methanol served as the control.

**Figure 5:**
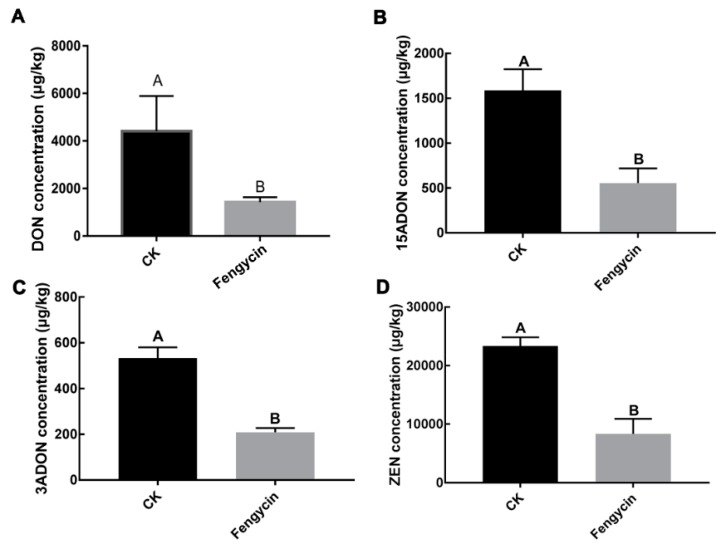
Effects of fengycin (90 µg/mL) on mycotoxins biosynthesis. Amount of DON (**A**), 15-ADON (**B**), 3-ADON (**C**), and ZEN (**D**) in infected wheat kernels 21 days after inoculation, with or without fengycin. Statistical analysis was carried out using Statistix 8.0 and subjected to one-way ANOVA with significant difference detected by Duncan’s multiple range test. Line bars denote standard errors of three replicate experiments and different letters describe significant differences at *p* < 0.01 within the same data group.

The results of fengycin application showed that fengycin could markedly reduce *F. graminearum* pathogenicity on wheat kernels (Figure 4). As DON is not only an important mycotoxin but also an essential virulence factor produced by *F. graminearum*, we further characterized the effect of fengycin on DON and other mycotoxins, i.e., 3-ADON,15-ADON, and ZEN biosynthesis in *F. graminearum*. The sterilized wheat kernels were incubated with *F. graminearum* and then treated with or without 90 μg/mL purified fengycin, and HPLC–MS analysis was performed to analyze mycotoxins production; we noted that fengycin could noticeably reduce DON, 3-ADON, 15-ADON, and ZEN biosynthesis. (Figure 5). Collectively, these results indicate that fengycin could reduce *F. graminearum* pathogenicity and mycotoxin biosynthesis.

## 3. Discussion

Apart from the chemical and conventional methods used for controlling diseases in plants, biological control is one of the safest and most effective alternative methods. Various microorganisms have been reported to be effectively used as a part of this method [15]. For example, *Bacillus* spp. act as effective antifungal and biocontrol organisms [16,17,18]. They produce many antifungal compounds, such as β-1,3-1,4-glucanase, chitinase, and lipopeptides [19,20,21]; such compounds can reportedly reduce or demine the activity of various phytopathogenic organisms.

Based on their structures, cyclic lipopeptides can generally be classified into four major families or groups: surfactin, iturin, fengycin, and locillomycin [22,23]. Fengycin, which has been isolated from members of the *Bacillus* genus, demonstrates strong antifungal activity and inhibits the growth of several plant pathogens, particularly of many filamentous fungi [4,24]. Likewise, fengycin antagonistically affected the growth of *F. graminearum* (FG-PH1) in vitro. *B. subtilis* has achieved a lot of attention as a biocontrol agent for manipulating many soil-borne diseases [25]. In this study, fengycin extracted from *B. amyloliquefaciens* FZB42 played a vital role in inhibiting growth of pathogenic fungus *F.* *graminearum.* Our studies have demonstrated that fengycin has an adverse effect on the structure of fungal hyphae and related activity of *F. graminearum* markedly decreases upon fengycin treatment [19,26,27,28]. The possible mechanism for the antifungal activity of fengycin is that it interacts with sterol and phospholipid molecules in the fungal cell membrane, altering its structure and permeability [29]. Our results, and even those of some previous studies, suggest that fengycin severely damages the plasma membranes and cell walls of *F. graminearum* hyphae and conidia, consequently causing cell death Fengycin influenced the cell membrane and cellular organs and also inhibited DNA synthesis [30,31]. It can also reportedly cause cell lysis of *M. fructicola* [31,32].

Current studies revealed that fengycin influences the pathogenicity of *F. graminearum* in plants. Fengycin caused a significant reduction in *F. graminearum* virulence, relative to the controls; the lengths of lesions on wheat spikes caused by *F. graminearum* markedly reduced upon treatment with fengycin. Similar results were also obtained in our previous studies when corn silk was treated with bacillomycin D [13].

Sporulation and germination play an important role in the asexual life cycle of *F. graminearum* and in spreading of FHB. In this study, fengycin strongly inhibited both the formation and germination of spores. Fengycin damaged the conidia and inhibited conidial germination; when spores of *F. graminearum* were used as inoculum to infect wheat heads, the severity of disease symptoms was reduced. In addition, considering the adverse effects of fengycin on fungal hyphae, it can inhibit the infection of corn silk by *F. graminearum* hyphae, as reported by a previous study [13]. Hence, the use of microorganisms producing antifungal compounds that inhibit conidial germination and hyphal growth should be an effective measure for biocontrol of fungal diseases.

Mitogen-activated protein kinase (MAPK) signaling pathways have been well characterized in *F. graminearum* [33,34]. Phosphorylation of the MAPKs FgHOG1 and FgMGV1 positively regulates environmental stress responses and DON biosynthesis [35,36]. Here, we observed that 90 μg/mL fengycin was enough to decrease mycotoxin biosynthesis in *F. graminearum*. In contradiction to former results, fengycin reduced the biosynthesis of secondary metabolites in *Fusarium graminearum*, while previous studies showed that the lipopeptides, such as Bacillomycin D and surfactin, induce significant fumonisin production in *F. verticillioides* [37]. Moreover, iturins reportedly induce HOG1 activation in *V. dahliae* [32]. Our results were similar to Kim et al., showing that the *B. amyloliquefaciens* JCK-12 has the ability to decrease both fungal growth and mycotoxin production. CLPs successfully inhibited DON production by affecting DON biosynthetic gene expression [38]. Our results also showed that the fengycin reduces the *F. graminearum* pathogenicity, as well as the most important virulence factor and mycotoxin biosynthesis in *Fusarium graminearum*. In summary, we report that fengycin produced by *Bacillus amyloliquefaciens* FZB42 has potential as a bio-control agent against wheat pathogen *Fusarium graminearum.* For future studies, other important derivatives of DON, such as deepoxy-deoxynivalenol, 3-epi-deoxynivalenol, or deoxynivalenol-3-glucoside, could also be studied under the influence of fengycin.

## 4. Materials and Methods

### 4.1. Bacterial and Fungal Strains Growth Conditions

In this study two previously constructed mutants, AK2 (Cannot synthesize fengycin) and AK1S (Can synthesize fengycin), from FZB42, were used [13] for activity against *F. graminearum* PH-1. Solidified Luria–Bertani medium was used to culture the mutants [39] and were activated in Landy medium [40]. Antibiotics ampicillin, chloramphenicol, and erythromycin were added at the following final concentrations: 100 g/mL, 5 g/mL, and 10 g/mL, respectively. For conidia formation fresh mycelia of PH-1 (50 mg) was taken from the margin of a 72 h grown colony and inoculated in 20 mL mung bean liquid medium [13], followed by incubation at 25 °C for 4 days in a shaker (180 rpm). A hemocytometer was used to count the number of conidia in each flask.

### 4.2. Anti-fungal Activity Assay

Antifungal activities of AK1S, AK2, and their secondary metabolite extracts were analyzed. Briefly, potato dextrose agar (PDA) was used to measure antifungal activity. A fungal block of 6-mm-diameter was patched at the middle of a PDA plate; 5 µl bacterial suspension (optical density at 600 nm of 2) or their secondary metabolite extracts were inoculated 3 cm away from the fungal block, and the plates were kept at 25 °C for 48 h; later, the diameters of the inhibition zones were measured.

### 4.3. Purification of Fengycin from AK1S and MALDI-TOF-MS Analysis

Fengycin was purified from AK1S as it could only produce fengycin; there was no possibility of contamination by either surfactin or bacillomycin D. A single colony of AK1S was inoculated into 20 mL LB medium and incubated for 18 h at 37 °C. Six milliliters of this culture was then inoculated in a 500-mL flask containing 200 mL of Landy medium, followed by incubation for 2 days at 30 °C. The sample was then centrifuged at 12,000× g for 20 min at 4 °C with Beckman Coulter Avanti J-26S XP centrifuge with JA-10 rotor (Beckman Coulter Brea, CA, USA) and the supernatant was collected. The pH of this cell-free extract was adjusted to 2, followed by centrifugation at 12,000× g for 20 min at 4 °C. This finally resulted in the precipitation of lipopeptides in the supernatant. Methanol was used to re-dissolve the precipitate, and the pH was adjusted to 7.0 using 1.0 M NaOH [41]. The supernatant was then passed through a silica gel column using different ratios of methanol and methylene chloride, termed MIX1 to MIX3 (methanol/methylene chloride ratios in MIX1, MIX2, and MIX3 were 1:2, 3:1, and 5:1, respectively). Preparative HPLC was performed for fengycin purification, in which we used a microbore 1100 HPLC system (Agilent Technologies, Santa Clara, CA, USA) with a VP 250/21 Nucleodur C18 HTec 5 µm column (Macherey-Nagel, Amtsgericht Düren, Germany); the eluates from MIX2 and MIX3 were collected and further used as mobile phase A, comprised of Acetonitrile with 0.1% (*v*/*v*) trifluoroacetic acid, and Milli-Q water with 0.1% (*v*/*v*) trifluoroacetic acid was used as mobile phase B. A solvent containing 45% mobile phase A and 55% mobile phase B was used at a flow rate of 8 mL/min for purification. For detection, UV absorption at 207 nm was recorded. The purity of fengycin collected from different peaks was detected using a 1200 HPLC system (Agilent Technologies, Santa Clara, CA, USA) with an Agilent Eclipse XDB-C18 5 µm column. Fengycin was confirmed by the appearance of one large peak at running times between 10 to 40 min. Purity of fengycin was calculated on the bases of the peak area (96.6%), which was used in our further studies. For antifungal activity of elution components from different peaks, they were tested and analyzed by MALDI-TOF-MS using a Bruker Daltonik Reflex MALDI-TOF instrument with a 337-nm nitrogen laser for desorption and ionization [42]. The α-Cyano-4-hydroxycinnamic acid served as the matrix.

### 4.4. Scanning Electron Microscopic Observation of Hyphal Morphologies

SEM was used to observe the morphological changes in *F. graminearum* hyphae caused by fengycin (90 µg/mL). To observe the fungal hyphae, they were treated with fengycin and 2.5% glutaraldehyde was used for prefixing. Subsequently, 100 mM phosphate buffer was used to rinse the fixed cells three times for 10 min; the samples were then post-fixed in 1% osmium tetroxide for 3 h and dehydration was done by using an ethanol gradient. Later, the samples were coated with gold particles and electron micrographs were obtained by using a Hitachi Science System Hitachi S-3000N scanning electron microscope at voltage 20 kV (H-7650, Hitachi, 251 Tokyo, Japan).

### 4.5. Plant Infection and Mycotoxin Production Assay

Wheat spikes were used to assess whether fengycin could adversely affect the pathogenicity of *F. graminearum*. When wheat reached the anthesis stage, the sixth spikelet from the base of the spike was pointed and inoculated with 10 µL of conidial suspension (10^5^ conidia/mL) containing 90 µg/mL fengycin. Only 10µl conidial suspension with 6.67% (*v*/*v*) methanol was used as the control. Three wheat spikes were inoculated for each treatment. The wheat plants were kept at 22 ± 2 °C and under 100% humidity for 2 days and then continued in a glass house. Diseased wheat kernels were examined and counted after 14 days. In order to ascertain the results, the experiment was repeated three times.

For the mycotoxin production assay, 50 g of healthy wheat spikes (wet weight) were surface sterilized by washing with 2% sodium hypochlorite and then inoculated with 1 mL of conidial suspension (10^6^ conidia/mL) containing 90 µg/mL fengycin and 6.67% (*v*/*v*) methanol, and incubated at 25 °C for 20 days. One milliliter of conidial suspension (10^6^ conidia/mL) with 6.67% (*v*/*v*) methanol served as the control. The experiment was repeated three times with three replicates for each. Mycotoxin extraction and quantification was performed by MycoSep 225 Trich Push Columns (Romer Laboratories Diagnostic (Getzersdorf, Austria) according to the manufacturer’s guidelines, avoiding the grinding of the starting material. The sample extract residue was dissolved in 400mL methanol/water (30:70). DON was quantified by HPLC according to the protocol followed by [43] with minor modifications. Separation was performed at room temperature by using a C18 reverse α-phase column (120 Å, 5 µm particle size, 4.66 × 150 mm, Acclaim) with an isocratic mobile phase of methanol/water (30:70) at a flow rate of 0.7 mL/min. Eluates were detected using a UV detector set at 220 nm. For quantification of DON, 3-DON, 15-Don, and ZEN, known amounts of pure standard bought from sigma were used as internal standards. Different concentrations of pure compound were analyzed by HPLC, and then the equation depicting the relationship between the concentration and HPLC peak area was obtained, which correlated the peak area to mycotoxin concentration [43].
